# Contrasting changes in extracellular dopamine and glutamate along the rostrocaudal axis of the anterior cingulate cortex of the rat following an acute *d*-amphetamine or dopamine challenge

**DOI:** 10.1016/j.neuropharm.2014.04.003

**Published:** 2014-12

**Authors:** Elizabeth S. Ash, David J. Heal, S. Clare Stanford

**Affiliations:** a Department of Neuroscience, Physiology and Pharmacology, University College London, Gower Street, London WC1E 6BT, UK; b RenaSci Ltd., Pennyfoot Street, Nottingham NG1 1GF, UK

**Keywords:** Anterior cingulate cortex, *d*-Amphetamine, Dopamine, Glutamate, GLT-1, Microdialysis, 5-HT, 5-hydroxytryptamine (serotonin), ACC, anterior cingulate cortex, cACC, caudal anterior cingulate cortex, AP, anteroposterior, Cg1, cingulate area Cg1, Cg2, cingulate area Cg2, Cg3, cingulate area Cg3, DV, dorsoventral, ECD, electrochemical detection, DA, dopamine, GLT-1, glutamate transporter 1, ML, mediolateral, mPFC, medial prefrontal cortex, NA, noradrenaline, rACC, rostral anterior cingulate cortex, VTA, ventral tegmental area

## Abstract

There is evidence for functional specificity of subregions along the rostrocaudal axis of the anterior cingulate cortex (ACC). The subregion-specific distribution of dopaminergic afferents and glutamatergic efferents along the ACC make these obvious candidates for coding such regional responses. We investigated this possibility using microdialysis in freely-moving rats to compare changes in extracellular dopamine and glutamate in the rostral (‘rACC': Cg1 and Cg3 (prelimbic area)) and caudal (‘cACC’: Cg1 and Cg2) ACC induced by systemic or local administration of *d*-amphetamine. Systemic administration of *d*-amphetamine (3 mg/kg, i.p.) caused a transient increase in extracellular dopamine in the rACC, but an apparent increase in the cACC of the same animals was less clearly defined. Local infusion of *d-*amphetamine increased dopamine efflux in the rACC, only. Glutamate efflux in the rACC was increased by local infusion of dopamine (5–50 μM), which had negligible effect in the cACC, but only systemic administration of *d*-amphetamine increased glutamate efflux and only in the cACC. The asymmetry in the neurochemical responses within the rACC and cACC, to the same experimental challenges, could help explain why different subregions are recruited in the response to specific environmental and somatosensory stimuli and should be taken into account when studying the regulation of neurotransmission in the ACC.

This article is part of the Special Issue entitled ‘CNS Stimulants’.

## Highlights

• Dopamine and glutamate efflux in two anterior cingulate subregions were compared.
• Responses to *d*-amphetamine depended on subregion and route of drug administration.
• These findings could help explain the disparate roles of the two subregions.

## 1 Introduction

The anterior cingulate cortex (ACC) lies within the medial prefrontal cortex (mPFC), which has a key role in governing cognition and associated behaviours. The existence of subdivisions of the ACC along its rostro-caudal axis is inferred from detailed cytochemistry (Jones et al., 2005 (rat); Vogt, 2005 (primate)), including mapping of its afferent inputs and efferent projections (Zeng and Stuesse, 1991, Zeng and Stuesse, 1993, Devinsky et al., 1995, Heidbreder and Groenewegen, 2003, Hoover and Vertes, 2007, Shibata and Naito, 2008). Functional specificity of these subregions has been confirmed in rodent lesion studies, human neuroimaging (Milad et al., 2007) and following brain trauma. For instance, there is a consensus that different subregions of the ACC process different aspects of pain sensation: whereas attention/perception seem to be processed in caudal regions in the rat, affective aspects of pain sensation are coded more rostrally (Devinsky et al., 1995, Bush et al., 2000, Johansen et al., 2001, Vogt, 2005). Characterisation of differences in neurotransmission in these subregions is important because they have been implicated in a range of disorders, including: Attention Deficit Hyperactivity Disorder (Bush et al., 1999), increased risk of psychosis (Fornito et al., 2008) and methamphetamine dependence (Paulus et al., 2003).

It has been suggested that there are reciprocal interactions between rostral and caudal zones of the ACC (Bush et al., 2000). This was borne out in studies that further suggest that reciprocal antagonism (‘anticorrelation’) between these zones extends to the subcortical brain regions to which each projects (Margulies et al., 2007, Fan et al., 2008). To date, there have been no comparisons of neurotransmission along the rostrocaudal axis of the ACC to explain such functional reciprocity.

There is a strong rationale for predicting that dopaminergic transmission, at least, will vary along the ACC. The dopaminergic innervation of the ACC is strikingly heterogeneous. The density of dopaminergic terminals is characteristically highest within Cg3 (now known as the prelimbic cortex) and declines in a caudal direction (van Eden et al., 1987). The Cg1/Cg2 regions of the ACC are innervated by dopaminergic neurones from both the A10 and A9 nuclei, but their distribution differs in superficial (mainly A9) and deep layers (mainly A10). By contrast, the prelimbic region is innervated by neurones projecting from A10, only (Emson and Koob, 1978, Lindvall et al., 1978, Swanson, 1982, Berger et al., 1985, Zeng and Stuesse, 1991). These two groups of neurones differ in a number of respects, such as: the extent of their collateralisation (Loughlin and Fallon, 1984), expression of dopamine transporters (greater in Cg1 than in Cg2 and prelimbic cortex: Freed et al., 1995, Sesack et al., 1998) and co-storage of neurotensin (Febvret et al., 1991).

As regards glutamate, it is well established that this transmitter influences dopamine release in the prefrontal cortex and elsewhere in the brain (PFC: *e.g.,* Feenstra et al., 1995, Del Arco and Mora, 2005). However, little is known about modulation of glutamate release by dopamine within the ACC. Yet, this interaction could have important functional consequences because dopaminergic terminals converge on cortical pyramidal cells, which are the conduit for cortical output. Dopamine also modulates pyramidal cell activity and glutamate release indirectly through interactions with both cortical GABA interneurons and glutamatergic neurones that project from subcortical regions, such as the thalamus and amygdala, to pyramidal cells in the prefrontal cortex (Berger et al., 1991, Seamans and Yang, 2004). The net effects of all these processes will have important consequences for cognition and behaviour because pyramidal cells in different zones of the ACC project to different subcortical brain regions in a series of cortico-subcortical circuits (Koski and Paus, 2000, Heidbreder and Groenewegen, 2003, Jones et al., 2005, Bissiere et al., 2008).

As a first step in characterising any differences in neurotransmission in subregions of the ACC that could underlie their functional reciprocity and specificity, we used *in vivo* microdialysis to compare changes in extracellular dopamine and glutamate (and their interaction) in a rostral and caudal region of the ACC following an acute *d*-amphetamine challenge. We chose this (pharmacological) stimulus because *d*-amphetamine increases the extracellular concentration of both dopamine and glutamate in the ACC and yet there has been no systematic investigation of the extent to which these responses generalise throughout its length.

The amount of dopamine and glutamate harvested by microdialysis probes (‘efflux’) was monitored in a rostral zone (rACC: spanning ventral Cg1 and the prelimbic (Cg3) area) and/or a caudal zone (cACC: incorporating the ventral zone of Cg1 and Cg2) of the ACC. These subregions are defined according to nomenclature in Paxinos and Watson (2005): but see Uylings et al., 2003) and were chosen because they are reported to differ functionally: in rats, the rACC is recruited in the response to glutamate-dependent conditioned avoidance learning, whereas the cACC responds to unconditioned noxious stimuli (Johansen et al., 2001).

Findings from this study point to striking differences in the regulation of dopaminergic and glutamatergic neurotransmission in the rACC and cACC. These differences echo reports of functional reciprocity between these subregions (Margulies et al., 2007) and could help govern their respective roles in cognition and affective behaviour.

## 2 Materials and methods

All procedures were carried out under the licensed authority of the UK Animals (Scientific Procedures) Act, 1986, and were approved by the local ethics committee at University College London.

### 2.1 Subjects and surgery

Male outbred Sprague–Dawley rats (250–300 g) were obtained from the colony at University College London. They were housed in groups of four, with sawdust bedding, at 21 ± 1 °C and 55 ± 1% relative humidity, with a light–dark cycle of 12 h (lights on at 08.00 h) and free access to standard laboratory chow and water. Anaesthesia was induced by inhalation of 5% halothane in 95% O_2_/5% CO_2,_ delivered at 2 L/min. Following loss of the righting reflex, rats were transferred to a stereotaxic frame and anaesthesia maintained *via* a face mask (2–2.5% halothane in 95% O_2_/5% CO_2_ at 1 L/min). The head was set in the flat-skull position (incisor bar set at 3.3 mm below the interaural line) using blunt ear-bars. Core temperature was maintained at 37 °C using a homeothermic heating pad and rectal probe.

**Fig. 1:**
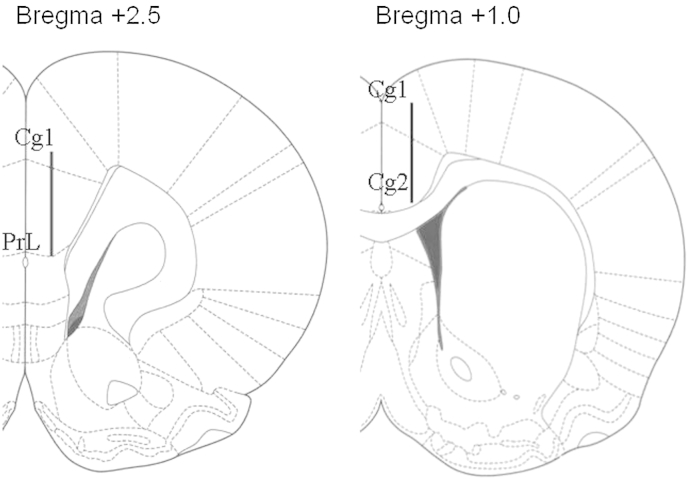
Typical locations of microdialysis probes (indicated by black lines) in the rACC (ventral Cg1 and prelimbic cortex) and cACC (Cg1 and Cg2) regions of the anterior cingulate cortex (adapted from Paxinos and Watson, 2005).

A small incision was made in the skin covering the skull. After trepanation, microdialysis probes, primed with modified Ringer's solution (NaCl, 145 mM; KCl, 4 mM; CaCl_2,_ 1.3 mM), were implanted either uni- or contralaterally (for single or dual-probe microdialysis, respectively) with the tip at the following co-ordinates (mm from Bregma): (rACC) AP +2.5, ML ±0.6, DV −4.6; (cACC) AP +1.0 ML ±0.6, DV -3.6 (Fig. 1, Paxinos and Watson, 2005). All probes were constructed in-house from Hospal (AN 69) dialysis membrane (see: Dalley and Stanford, 1995) and were equipped with a dialysis window that extended 2 mm above the end of the probe. After anchoring the shaft of the probe(s) to the skull with acrylic cement, the rats were allowed to recover from the anaesthesia, with lignocaine local anaesthetic cover, in an incubation chamber, before transfer to individual plastic cages. On the following day, the inlet and outlet tubes were guided through a liquid swivel, to enable the rat to move freely around the cage, and the probes perfused with the modified Ringer's solution at a rate of 2 μL/min. Dialysis samples (40 μL) were collected every 20 min and experiments were started after a minimum of three successive samples confirmed stable (baseline) transmitter efflux. In experiments studying the response to local infusion of *d*-amphetamine or dopamine, different microsyringes were used to deliver each of the test solutions. To minimise any spontaneous degradation of the solutes, they were loaded onto the syringe pump immediately before use and connected to the inflow for the microdialysis probe with minimal disturbance to the animal. At the end of each experiment, the animals were deeply anaesthetised and killed by cervical dislocation. Brains were removed and stored overnight in formalin solution for verification of probe placement.

### 2.2 Experimental protocols

***Experiment 1:*** Probes were implanted contralaterally for simultaneous microdialysis of the rACC and the cACC. The next day, spontaneous (basal) efflux of dopamine was monitored in both subregions until it had stabilised, after which three basal samples were designated *T*_−__40_-*T*_0._ At *T*_0_, rats were randomly assigned to groups given either *d-*amphetamine (3 mg/kg, i.p), or saline (1 mL/kg): these two treatments were administered in a counterbalanced sequence. This dose of *d-*amphetamine was chosen because it is known to increase dopamine efflux without inducing stereotypy (Segal and Mandell, 1974) and is within the range of those used typically to study its effects on dopaminergic transmission (*e.g.,* Goodwin et al., 2009).

In a different batch of rats, dopamine efflux was monitored during local infusion of *d-*amphetamine into the rACC and cACC, *via* the microdialysis probe (10 μM *d*-amphetamine for 2 h, followed by modified Ringer's solution for 1 h, and then 100 μM *d-*amphetamine for 2 h). These concentrations were used because they increase dopamine efflux in other brain regions (*e.g.,* Mazei et al., 2002) and they are within the range of (or lower than) those used in similar published studies *in vivo* (Ventura et al., 2004, Géranton et al., 2003a, Géranton et al., 2003b, Abercrombie and De Boer, 1997 and further references cited therein). It should be borne in mind that the concentration of *d*-amphetamine in the extracellular fluid will be maximum immediately adjacent to the probe but will fall progressively as distance from the probe increases. As a consequence, the concentration of *d-*amphetamine within the aura of the probe will span not only those used routinely to study its actions *in vitro* (*e.g.,* Pifl et al., 1995, Del Arco et al., 1998, Bosse et al., 2008) but also the concentrations found typically in human plasma in pharmacokinetic studies of this drug (Westerink and De Vries, 2001, Asghar et al., 2003, Jasinski and Krishnan, 2009).

***Experiment 2***: Microdialysis probes were implanted in either the rACC or the cACC of pairs of rats to compare glutamate efflux in the two subregions following a *d*-amphetamine challenge. The rats were then randomly assigned to one of two test groups. One was given *d-*amphetamine by systemic injection (3 mg/kg i.p.) at *T*_0_. This dose is the same as that in Experiment 1 and has been reported to increase glutamate efflux in the rostral zone of the medial prefrontal cortex (Reid et al., 1997). The second group experienced local infusion of *d-*amphetamine: 1 μM (80 min), followed by 10 μM (80 min) and then 100 μM (160 min).

***Experiment 3***: Probes were implanted on contralateral sides of the brain for dual-probe microdialysis of the rACC and the cACC. The probes were used both to harvest extracellular glutamate and for local infusion of dopamine. The stock dopamine solution was kept on ice, in the dark, until loading into syringes for simultaneous infusion into both the rACC and cACC. The test concentrations of dopamine (0.05, 0.5, 5 and then 50 μM (80 min each)) were similar to those used previously (Pan et al., 2004). There was no correction for spontaneous degradation of dopamine, which would be the same for both regions.

### 2.3 Chromatography

Immediately after collection of the microdialysis samples, glutamate (which was analysed after precolumn derivatisation with 20 μL of complete *o-*phthalaldehyde reagent (Rowley et al., 1995)) and dopamine were separated by HPLC and measured by electrochemical detection (ECD) using an ESA Coulochem II detector. Both HPLC systems incorporated an Aquapore (7 μM) guard column but different main columns (both maintained at 26 °C) were used to separate dopamine (Hypersil ODS; 5 μM; 250 × 4.6 mm) and glutamate (Supercosil™LC-18; 5 μM; 150 × 4.6 mm). For glutamate, the mobile phase comprised (mM): Na_2_HPO_4_ 64, NaH_2_PO_4_ 16, EDTA 0.13, dissolved in 25% methanol, adjusted to pH 6.5 with phosphoric acid and pumped through the system at 1 ml/min. The conditioning electrode of the microdialysis cell (ESA 5014A) was set at −280 mV and the analytical electrode set at +350 mV. For dopamine, the mobile phase comprised (mM): NaH_2_PO_4_ 83, octanesulphonic acid (OSA) 0.23, EDTA 0.84, dissolved in 17% methanol, adjusted to pH 4.0 with orthophosphoric acid and pumped through the system at 1.15 ml/min. The conditioning electrode of the microdialysis cell (ESA 5014A) was again set at −280 mV but the analytical electrode was set at +180 mV. The yield of solutes is expressed as fmol/20 min (dopamine) or pmol/20 min (glutamate), with no adjustment for probe recovery. The limit of detection was 1 pmol (glutamate) and 2–4 fmol (dopamine). In two rats, the dopamine content of some baseline samples was below the limit of detection and was assigned a value of 0 fmol/20 min: both were from the group serving as saline-injected controls for the effects of systemic administration of *d*-amphetamine on dopamine efflux in the rACC (Experiment 1). Since there was no dopamine response in these animals, this will not affect the interpretation of the results. There were no animals with basal DA levels below the limit of detection in the local infusion experiments.

### 2.4 Statistical analysis

For each animal, mean basal efflux was calculated from the three consecutive samples taken immediately before the experimental challenge. These data were pooled to provide a measure of mean basal efflux for each experimental group. The significance of differences in transmitter efflux was assessed using multi-factorial split-plot ANOVA (SPSS PC) with repeated measures on the factor, ‘time’. ANOVA was carried out on the raw data unless the Mauchly test indicated statistically significant deviation from homogeneity of the variance/covariance matrix on the repeated measures factor (time). In those cases, ANOVA was carried out on log_(10)_ transformed data, provided this reduced statistical significance in the Mauchly test. When significance in this test remained, despite the transformation, the Greenhouse-Geisser *ε* correction was applied to the degrees of freedom. The Levene's test was applied to ensure equal variances across all levels of the between subjects' factors in the log_(10)_transformed data set. In all cases, a significant effect on the main factor(s), or interactions between them, was taken as the criterion for progressing to a *post-hoc* 2-way ANOVA. In experiments testing the effects of systemic drug administration, the data were divided into clusters (‘bins’) of consecutive samples. The bins comprised 3 samples (*i.e.,* 1 h of sampling time) in order to assess the significance of any changes in transmitter efflux. In experiments involving local infusion of *d-*amphetamine or dopamine, the bins corresponded to samples collected during infusion of each test concentration (*i.e.,* 80 min). In these cases, only the last 3 samples in each bin were compared with the 3 basal samples and ‘bin’ was treated as another within-subjects’ factor in the ANOVA. ‘Subregion’ was treated as a ‘between-subjects’ factor in single-probe microdialysis and a ‘within-subjects’ factor in the dual-probe experiments. The criterion for significance was *P* ≤ 0.05.

### 2.5 Drugs and reagents

*d*-Amphetamine sulphate and dopamine hydrochloride were obtained from Sigma–Aldrich (UK). When given by intraperitoneal injection, the drug was dissolved in 0.9% sterile saline and administered in a volume of 1 mL/kg. When infused locally into the terminal field, *d-*amphetamine was dissolved in the modified Ringer's perfusion solution. All reagents for the modified Ringer's solution and mobile phase were of chromatographic or AnalaR grade.

## 3 Results

There was an increase in locomotor activity after systemic injection of *d-*amphetamine but not during its local infusion. We saw no sign of behavioural stereotypy in either case.

### 3.1 Experiment 1: the dopamine response to d-amphetamine depends on subregion of the ACC and the route of drug administration

**Fig. 2:**
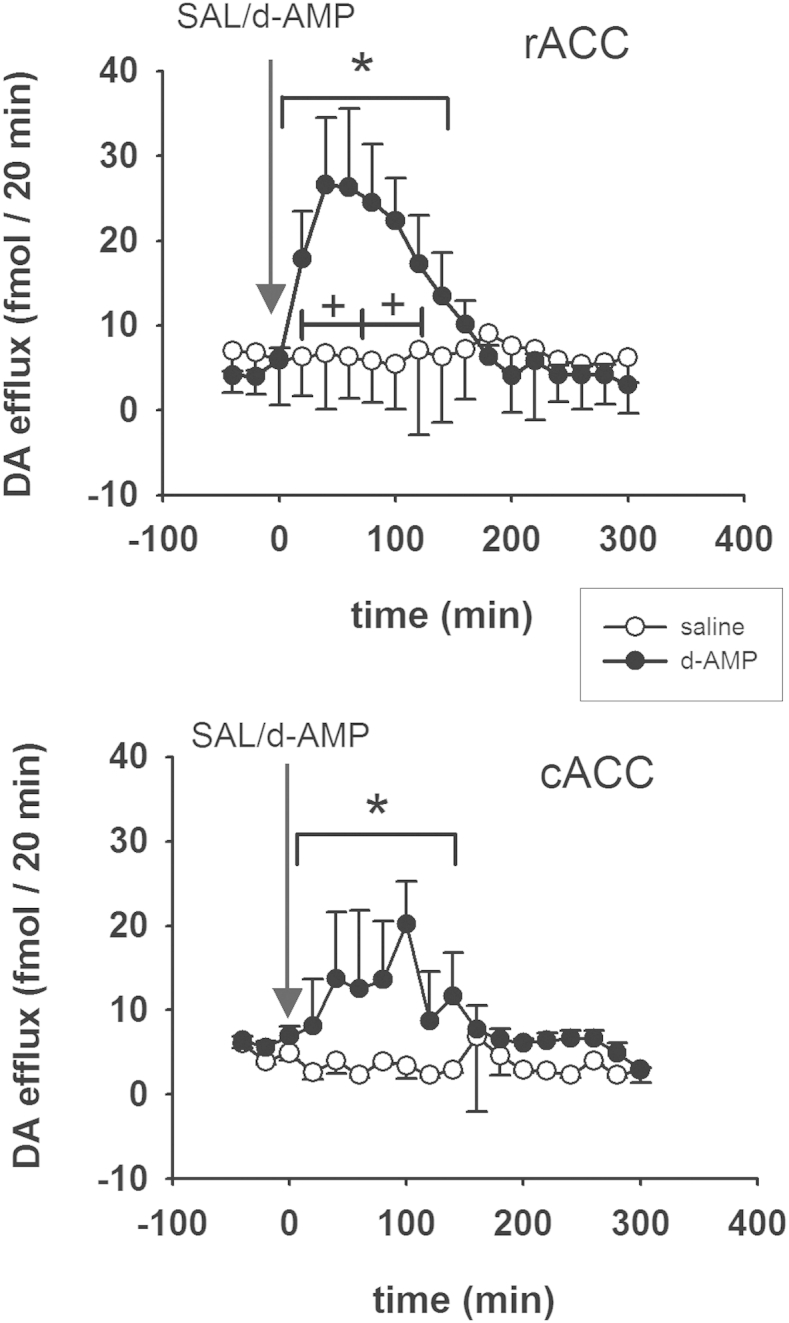
Effect of systemic administration of *d*-amphetamine (‘*d*-AMP’, 3 mg/kg, i.p) or saline (1 mL/kg) on dopamine (‘DA’) efflux in the rACC or cACC of freely-moving rats. Animals were injected at *T*_0_, indicated by the arrow. DA efflux is expressed as fmol/20 min. Points show mean ± s.e. mean. *N* = 4-6 in each group. Statistical analysis was carried out on Log_(10)_ transformed data and revealed an increase in DA efflux in both subregions between *T*_20_ and *T*_140_. *: *P* < 0.05 (*c.f., d*-amphetamine/saline) over the time-period indicated by the bar. +: *P* < 0.05 *c.f*., time-matched samples from the two groups (*T*_−40_–*T*_0_) for the bins indicated by the bar.

There was no difference in mean basal efflux of dopamine in the rACC [4.4 ± 0.4 fmol/20 min] and cACC [5.7 ± 0.8 fmol/20 min] of animals destined for intraperitoneal injection of saline or *d*-amphetamine (3 mg/kg). Saline injection did not affect dopamine efflux in either region (Fig. 2). Dopamine efflux increased in the rACC after injection of *d-*amphetamine: this response was evident within 20 min of *d*-amphetamine administration, reached a maximum of 27 ± 8 fmol/20 min at *T*_40,_ and lasted for approximately 2 h [*c.f. d*-amphetamine/saline (*T*_20_–*T*_140_): *F*(1,9) = 5.8; *P* = 0.04; *ε* = 0.45]. This increase was also evident when efflux after drug administration was compared with the basal samples [1st hour: *F*(1,4) = 10.4, *P* < 0.03; 2nd hour: *F*(1,3) = 10.0, *P* < 0.05]. In the cACC of the same animals, the increase in dopamine efflux also reached criterion for statistical significance when compared with the saline group [*T*_20_–*T*_140:_ *F*(1,7) = 10.3; *P* = 0.015; *ε* = 0.344]. However, unlike the rACC, the increase in dopamine efflux did not reach criterion for statistical significance when compared with the basal samples. The response to *d*-amphetamine in the cACC was also more erratic than in the rACC, but there was no difference in dopamine efflux in the two subregions within this time-frame [*F*(1,16 = 1.384; *P* = 0.257] and no interaction between drug treatment and subregion [*T*_20_–*T*_140:_ *F*(1,16) = 0.025; *P* = 0.88] (Fig. 2).

**Fig. 3:**
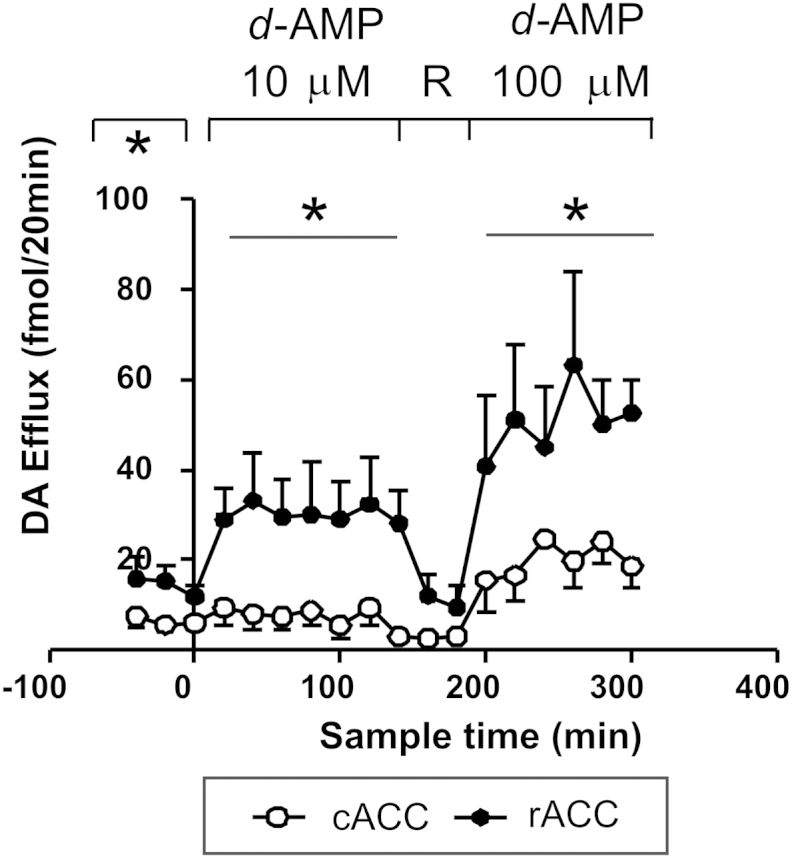
Dopamine (DA) efflux during local infusion (*via* retrodialysis) of *d*-amphetamine (‘*d*-AMP’) into the rACC or cACC of freely-moving rats. *d*-Amphetamine was infused from *T*_0_–*T*_120_ (10 μM) and from *T*_180_–*T*_300_ (100 μM) (indicated). R = Ringer's solution. *N* = 7. DA efflux is expressed as fmol/20 min. Points show mean value ± s.e. mean. *: *P* < 0.05 (rACC: *c.f.,* cACC) for the period indicated by the bar. Both concentrations of *d*-amphetamine increased dopamine efflux in the rACC but not the cACC.

In a second cohort of rats, destined for local infusion of *d*-amphetamine, the mean basal efflux of dopamine in the rACC [14.3 ± 2.4 fmol/20 min] was greater than in the cACC [6.4 ± 1.4 fmol/20 min] [*F*(1,11) = 7.69; *P* = 0.02] (Fig. 3). The dopamine response depended strongly on subregion such that, during infusion of *d*-amphetamine, overall dopamine efflux was greater in the rACC than the cACC [*F*(1,17) = 15,67; *P* < 0.001; *ε* = 0.623]. Specifically, infusion of the lower concentration of *d-*amphetamine (10 μM) increased dopamine efflux in the rACC but not the cACC [*c.f.,* rACC and cACC; *T*_20_–*T*_120_: *F*(1,10) = 9.53; *P* = 0.012; *ε* = 0.55] (Fig. 3). Dopamine efflux returned to basal levels when the modified Ringer's perfusion solution was reinstated. When the higher concentration of *d*-amphetamine was substituted for the modified Ringer's solution (100 μM), there was a resurgence of dopamine efflux in the rACC, which reached maximum at *T*_260_ [63 ± 21 fmol/20 min]. Again, dopamine efflux in the rACC was greater than in the cACC [*c.f.,* rACC and cACC; *T*_200_–*T*_300_: *F*(1,7) = 8.94; *P* = 0.02; *ε* = 0.36]. The apparent increase in the cACC at the higher concentration of *d-*amphetamine did not meet the criterion for statistical significance [*c.f*., *T*_20_–*T*_120_ and *T*_200_–*T*_300_; *F*(1,8) = 2.44; *P* = 0.11; *ε* = 0.47] (Fig. 3).

### 3.2 Experiment 2: the glutamate response to d-amphetamine depends on subregion of the ACC and route of drug administration

Mean basal efflux of glutamate in the rACC and cACC was 10.4 ± 1.8 pmol/20 min and 9.1 ± 1.7 pmol/20 min, respectively, and did not differ in animals destined for intraperitoneal injection or local infusion of *d*-amphetamine.

**Fig. 4:**
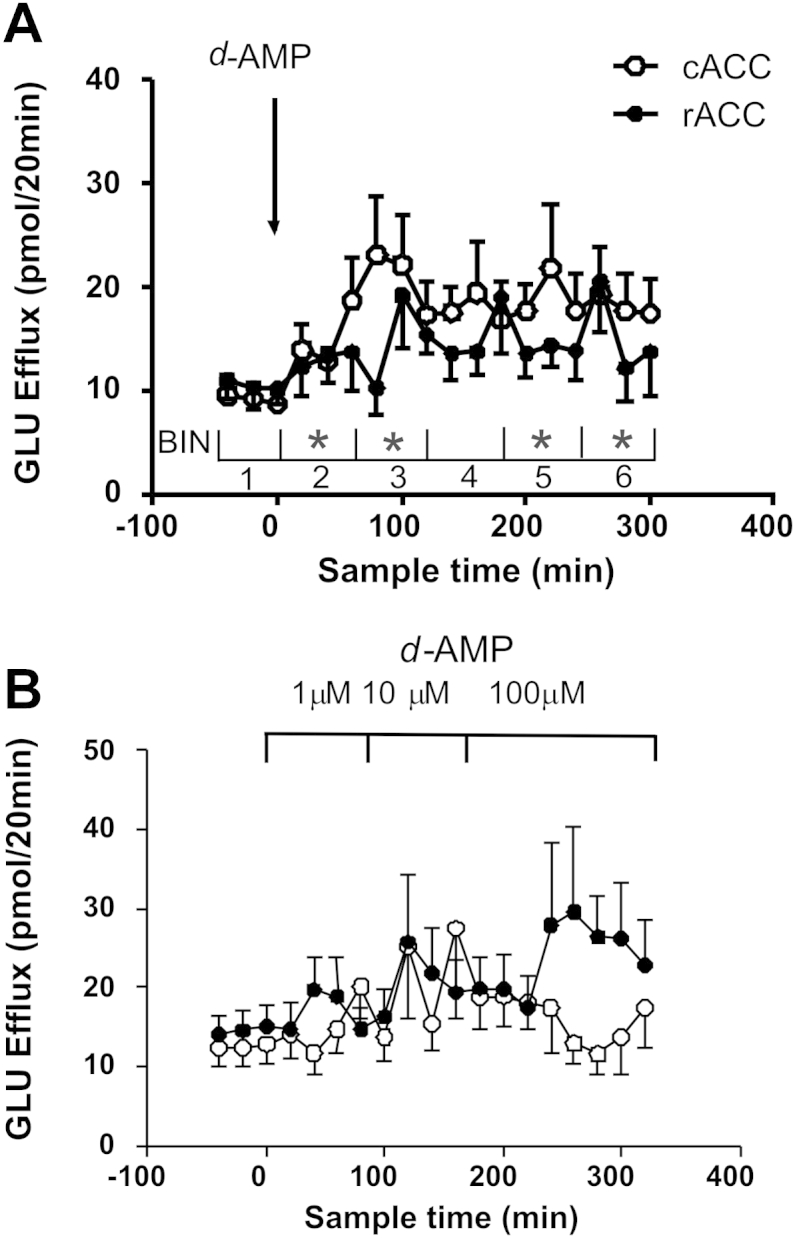
Glutamate (‘GLU’) efflux (A) following systemic administration of *d*-amphetamine (‘*d*-AMP’: 3 mg/kg, i.p) at *T*_0_ (indicated by arrow) (*N* = 10). For analysis, data were divided into 6 time bins of 3 samples, as indicated. Points show mean ± s.e. mean. *: *P* < 0.05 (cACC: *d*-AMP *c.f*. basal samples in Bin 1) (B) during local infusion (*via* retrodialysis) of *d-*amphetamine at the concentrations and times indicated (*N* = 7). GLU efflux is expressed as pmol/20 min. Points show mean ± s.e. mean.

Following systemic administration of *d*-amphetamine (3 mg/kg, i.p.), there was no statistical interaction between subregion and glutamate response (Fig. 4A). Nevertheless, it is striking that glutamate efflux in the rACC seemed to oscillate more than that in the cACC and, when compared with the basal samples, did not reach the criterion for statistical significance at any time. By contrast, in the cACC, there was a robust and more stable increase in glutamate efflux within the first hour post-injection [*c.f*., *T*_40_–*T*_80_ (bin 2) and the basal samples (T_-40_–*T*_0_): *F*(1,16) = 6.21; *P* = 0.024; *ε* = 0.929]. The maximum increase occurred at *T*_80_ [+11.1 ± 7 pmol/20 min]. Apart from the samples collected from *T*_160_–*T*_200_ [(bin 4): *c.f.,* basal samples: *F*(1,17) = 4.34; *P* < 0.08; *ε* = 0.777], efflux remained greater than that in the basal samples for the remainder of the experiment [*c.f.,* bin and the basal samples; *T*_100_–*T*_140_ (bin 3): *F*(1,17) = 6.554; *P* < 0.02; *ε* = 0.756; *T*_220_–*T*_260_ (bin 5): *F*(1,18) = 6.06; *P* < 0.02; *ε* = 0.659; *T*_280_–*T*_320_ (bin 6): *F*(1,17) = 6.85; *P* < 0.018; *ε* = 0.537] (Fig 4A).

Local infusion of *d*-amphetamine did not increase glutamate efflux in either subregion. The apparent increase in glutamate efflux into the rACC during infusion of 100 μM *d*-amphetamine [*T*_260_–*T*_320_], was not statistically significant when compared with either the basal samples or across the two regions (Fig. 4B) (even after removal of an outlier (>(3 × standard deviations) from mean).

### 3.3 Experiment 3: local infusion of dopamine increases glutamate efflux in the rACC but not the cACC

**Fig. 5:**
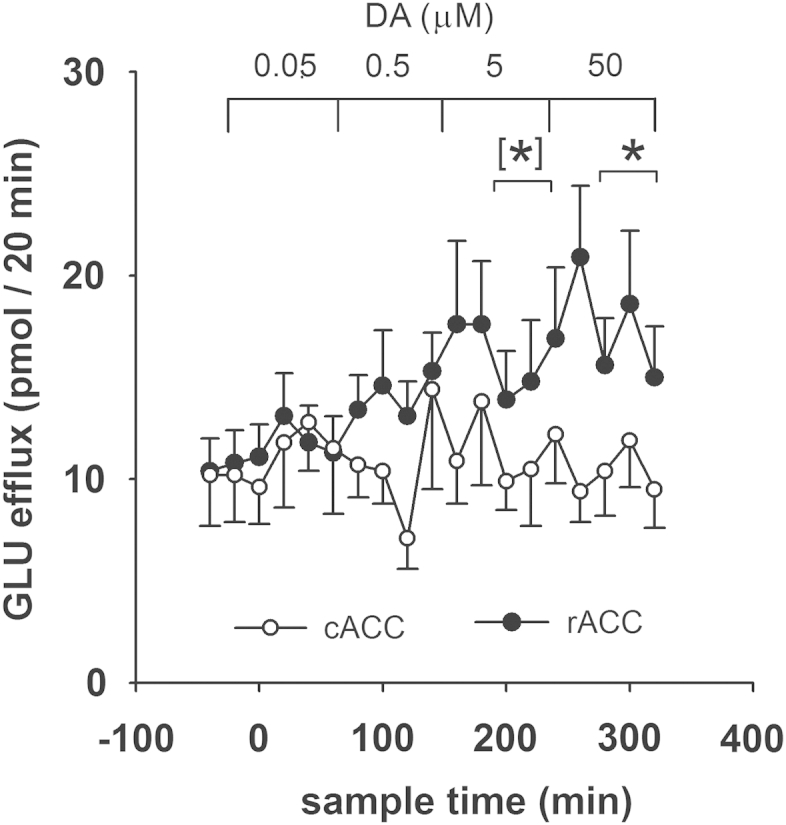
Effects of local infusion of dopamine (‘DA’) *via* retrodialysis, at the concentrations (μM) and times indicated, on glutamate (‘GLU’) efflux in the rACC and the cACC of freely-moving rats. Dopamine infusion started at *T*_0_. GLU efflux is expressed as pmol/20 min. Points show mean value ± s.e. mean. *N* = 12–14. For statistical analysis, the data were divided into 5 time-bins of 3 samples. The first samples of each bin, taken 20 min after the change in perfusion solution, were not included in the analysis. *: *P* < 0.05, [*]:*P* = 0.059 *c.f.,* time-matched samples in the two brain regions.

Local infusion of dopamine increased glutamate efflux in the rACC but not the cACC. Over the entire series of samples, an interaction between concentration of dopamine infusion and brain region just missed criterion for statistical significance [*F*(1,17) = 4.19; *P* = 0.056; *ε* = 0.28]. However, efflux in the rACC during infusion with 50 μM dopamine was greater than in time-matched samples from the cACC [*F*(1,22) = 4.41; *P* = 0.048; *ε* = 0.926] and the response to 5 μM dopamine in the rACC just missed the criterion for significance [5 μM DA: *F*(1,25) = 3.91, *P* < 0.059; *ε* = 0.883] (Fig. 5).

## 4 Discussion

The results of this study reveal striking differences in dopaminergic and glutamatergic responses in the rACC and the cACC following a *d*-amphetamine challenge.

The first experiments used dual-probe microdialysis to monitor dopamine efflux, simultaneously, in these two subregions. Either systemic administration or local infusion of *d-*amphetamine produced a prominent dopamine response in the rACC. Systemic administration, but not local infusion of *d*-amphetamine, also caused a marginal increase in dopamine efflux in the cACC. It is possible that a higher concentration of local *d*-amphetamine would have provoked a dopamine response in the cACC. However, the key finding from these experiments was that local administration of *d*-amphetamine had negligible effects on dopamine efflux in the cACC at concentrations that were effective in the rACC of the same animals.

Several factors could contribute to the more resilient response to *d-*amphetamine in the rACC (see also: Pehek, 1999), notably when administered locally. One is the greater density of dopaminergic terminals, especially in the prelimbic region (van Eden et al., 1987). Another is the relatively low density of dopamine transporters in the prelimbic region, compared with other regions of the prefrontal cortex (see: Sesack et al., 1998 (and references cited therein); Mundorf et al., 2001). This would constrain dopamine clearance, which is a major determinant of the concentration of extracellular transmitter (Smith and Justice, 1994, Géranton et al., 2003a). Moreover, accumulation of extracellular dopamine will be augmented by *d-*amphetamine because this compound is a substrate for translocation by the dopamine transporter, and so a competitive inhibitor of dopamine uptake, as well as a dopamine releasing-agent (at high concentrations). There is also evidence that dopamine transporters in the prelimbic cortex are concentrated in intervaricose regions of the nerve terminal (Sesack et al., 1998), which would further reduce clearance efficiency and increase the amount of dopamine harvested by the microdialysis probe. Given the evidence for ectopic uptake of dopamine by noradrenaline transporters in the superficial medial prefrontal cortex (Cass and Gerhardt, 1995), another possible explanation is that this process is more vigorous in the cACC than the rACC. Also, somatodendritic (inhibitory) autoreceptors on neurones projecting from the substantia nigra (A9), which could be activated directly or indirectly (*via* polysynaptic loops), have a greater inhibitory influence on neuronal firing-rate than those in the ventral tegmental nucleus (A10) (Cragg and Greenfield, 1997). This range of mechanisms, which would influence the concentration of extracellular dopamine after treatment with *d*-amphetamine, all tend to *increase* dopamine efflux in the rACC but to *constrain* it in the cACC.

Regional variation in the dopamine response to *d*-amphetamine could also rest on differences in factors such as: terminal heteroceptors that govern dopamine release in the anterior cingulate cortex (Gobert et al., 1998); the dimensions of the synaptic space; tortuosity of the extrasynaptic space; uptake by the organic cation transporter 3 (Gasser et al., 2009). Region-specific inhibition of impulse-evoked dopamine release, mediated by glutamatergic neurones, is also possible. For instance, glutamatergic influences on dopaminergic neurones within the ventral tegmental nucleus can either augment or inhibit impulse-dependent dopamine release in the terminal field; this process has been proposed to underlie functional heterogeneity of dopaminergic projections to different brain regions (Takahata and Moghaddam, 1998; see also: Del Arco and Mora, 2009). Differences in any of these disparate pharmacokinetic and pharmacodynamic influences on transmitter efflux could account for the greater *d-*amphetamine-induced increase in dopamine efflux in the rACC, compared with the cACC, even in the absence of a difference in basal efflux.

These empirical observations prompted us to investigate whether the net effect of all these processes has any bearing on glutamatergic transmission within these subregions. The effect of *d*-amphetamine on glutamate efflux, like dopamine, depended on subregion of the ACC. Once again, route of administration was an important factor, also. Clearly, we cannot assume that the concentration of extracellular *d-*amphetamine in the ACC in these studies was the same after its systemic administration and local infusion. Nevertheless, our findings indicate that although systemic administration of *d*-amphetamine at this dose (3 mg/kg) caused a clear increase in glutamate efflux in the cACC, this was not the case for the rACC.

So far, no study has compared directly the glutamate response to the same experimental challenge in different zones of the rostrocaudal axis of the ACC. We cannot distinguish whether the response to systemic drug treatment, alone, accounts for the regional difference in the glutamate response or whether a response to drug injection is a contributing factor. Nevertheless, the key finding is that the glutamate response to local infusion of dopamine or systemic administration of *d*-amphetamine differs in the two subregions. These findings are supported by published evidence to the extent that glutamate efflux in the rACC is not increased by systemic administration of *d*-amphetamine (*c.f.,* AP: +3.2; Shoblock et al., 2003).

The explanation for these regional differences in the glutamate response is unclear, not least because there are several possible sources of glutamate release from neurones in the ACC. These include: dendrites of cortical pyramidal cells; terminals of cortico-cortical pyramidal collaterals; and glutamatergic afferents from subcortical regions (thalamocortical and amygdala) that converge on somatodendritic regions of cortical pyramidal cells, which, together with the terminals of dopaminergic afferents from A9 and A10 nuclei, form neuronal ‘triads’ (Berger et al., 1991). Also, as discussed later, glial cells are thought to serve as the prominent source of glutamate harvested by microdialysis probes.

Both dopamine D1-like receptors and, to a lesser extent, D2-like receptors are expressed by pyramidal cells. The density of these receptor subtypes varies with cortical subregion but is comparatively high in the prelimbic region. It is now generally agreed that these receptors have no consistent effect on pyramidal cell function: in electrophysiological studies *in vitro*, the pyramidal cell response to activation of dopamine receptors is time- and concentration-dependent; it can be mediated both directly and indirectly; and depends on pyramidal cell excitable state (see: Seamans and Yang, 2004, Santana et al., 2009). Nevertheless, obvious questions that arise from our findings are whether dopaminergic transmission augments glutamate efflux in the rACC but has little, if any, effect in the cACC, and whether this has any bearing on the different glutamate responses to *d*-amphetamine in the two subregions.

On the basis that the increase in glutamate efflux on local infusion of dopamine was confined to the rACC, it is unlikely that dopaminergic transmission within the cACC mediates the glutamate response to systemic administration of *d*-amphetamine. However, *d*-amphetamine also evokes release of noradrenaline and serotonin, which could be alternative candidates for mediating the glutamate response. Noradrenaline is likely to be of greater importance because the density of noradrenergic terminals in the prelimbic cortex is higher than in other cingulate areas (Morrison et al., 1979). Also, the concentration of extracellular noradrenaline is increased in the rACC by both local and systemic administration of *d*-amphetamine (Géranton et al., 2003b). By contrast, *d*-amphetamine causes a relatively small increase in serotonin release (Pum et al., 2007) and neither systemic injection nor local infusion of the serotonin releasing-agent, dexfenfluramine, increases extracellular glutamate in the rostral frontal cortex (Rocher et al., 1996).

However, the lack of any glutamate response to local infusion of *d-*amphetamine or dopamine within the cACC suggests that the increase in glutamate efflux in this region, following systemic administration of *d*-amphetamine, must recruit processes beyond the aura of the probe. These could include distant neurones within the cACC (*i.e.,* terminals of collaterals from remote ipsilateral/contralateral cortical pyramidal cells) but polysynaptic links are possible, also, such as indirect activation of glutamatergic thalamocortical neurones.

It is important to bear in mind that glial cells (microglia and astrocytes) are a prominent source of extracellular glutamate, which is extruded on a glial cystine/glutamate antiporter (Baker et al., 2002). This source of glutamate could be particularly important in our experiments in view of evidence that glutamate harvested by microdialysis probes, is non-synaptic in origin (Timmerman and Westerink, 1997, van der Zeyden et al., 2008). Glial cells also display Ca^2+^-dependent exocytosis of glutamate (Montana et al., 2006; reviewed by Malarkey and Parpura, 2008). Given that astrocytes can be activated by dopamine (Reuss and Unsicker, 2001) and influence the activity of neighbouring neurones, they could have a key role in the regulation and integration of ACC function (Del Arco et al., 2003, Zhang and Haydon, 2005; reviewed by Fillenz, 2005). Indeed, *d*-amphetamine and dopamine are already known to induce glial cell activation in the striatum (Thomas et al., 2004) and extrusion of glutamate on the glial transporter, GLT-1, in the ventral tegmental area (Wolf et al., 2000). We are currently investigating the possibility that the difference in *d*-amphetamine-evoked glutamate efflux in the rACC and cACC involves glial cell activation and/or glutamate clearance on GLT-1 transporters.

In summary, this study has revealed a marked asymmetry in the dopaminergic and glutamatergic responses within the rACC and the cACC following an acute *d*-amphetamine or dopamine challenge. Such regional disparities should be taken into account in electrophysiological and neurochemical studies of the ACC *in vivo* and *in vitro*. Moreover, these contrasting responses could contribute to the ‘functional reciprocity’ of subregions of the ACC (*e.g.,* Margulies et al., 2007), enabling them to make different contributions to the regulation of mood and behaviour.

## Sources of support

EA was in receipt of a Medical Research Council (UK) PhD studentship. This work was funded by the 10.13039/501100000779University of London (Central Research Fund) and RenaSci Ltd. The funders were not involved in defining the study objectives, the experimental design, interpretation of the results, or drafting of this paper.
